# Comparative Analysis of C-reactive Protein and Procalcitonin as Biomarkers for Prognostic Assessment in Pediatric Sepsis

**DOI:** 10.7759/cureus.65427

**Published:** 2024-07-26

**Authors:** Neha Tyagi, Siddhi Gawhale, Manojkumar G Patil, Sampada Tambolkar, Shradha Salunkhe, Shailaja V Mane

**Affiliations:** 1 Pediatrics, Dr. D. Y. Patil Medical College, Hospital and Research Centre, Dr. D. Y. Patil Vidyapeeth (Deemed to be University) Pimpri, Pune, IND

**Keywords:** prognostic utility, biomarkers, pediatric, procalcitonin, c-reactive protein, sepsis

## Abstract

Background

Sepsis poses a critical medical challenge due to its profound systemic inflammatory response, which frequently results in organ dysfunction and high mortality rates, especially in pediatric patients. The condition requires prompt recognition and aggressive management to mitigate its severe outcomes.

Methods

This prospective study enrolled 248 pediatric patients admitted with sepsis to the pediatric intensive care unit (PICU) at our tertiary care center. Patients were randomly assigned to either the C-reactive protein (CRP) or procalcitonin (PCT) groups, with biomarker levels measured upon admission (hour zero) and again at 72 hours post-admission. Clinical parameters such as the need for ionotropic support, use of steroids, incidence of acute kidney injury (AKI), requirement for invasive ventilation, patient outcomes, and changes in antibiotic management were assessed based on these biomarker levels.

Results

Procalcitonin-positive sepsis cases demonstrated notable clinical severity compared to their C-reactive protein counterparts, showing significantly lower systolic blood pressure (p = 0.012), heightened need for ionotropic support (p < 0.0001), and more pronounced liver and renal dysfunction as indicated by elevated serum bilirubin (p = 0.001) and creatinine levels (p = 0.0058). The incidence of AKI was also higher in procalcitonin-positive cases. Despite these severe clinical parameters, there were no significant differences in the length of the PICU stay or in patient outcomes concerning discharge and mortality rates. Procalcitonin levels effectively guided antibiotic management, resulting in therapy adjustments in a substantial proportion of cases, with 67 (54%) experiencing downgrades and 33 (27%) requiring upgrades based on procalcitonin levels measured 72 hours post-admission.

Conclusion

Procalcitonin proves to be a valuable biomarker in assessing the severity and management of sepsis in pediatric patients. It correlates significantly with clinical parameters such as blood pressure, the need for ionotropic support, and markers of organ dysfunction.

## Introduction

Severe sepsis, potentially a life-threatening condition caused by the body's exaggerated reaction to infection, is particularly problematic in the pediatric population [1]. The systemic inflammatory response associated with sepsis often leads to multi-organ dysfunction and significant mortality rates, making early diagnosis and effective management crucial [2]. The need for reliable biomarkers to predict sepsis severity and guide therapeutic interventions has spurred considerable research in this field [3]. C-reactive protein (CRP) and procalcitonin (PCT) are widely recognized biomarkers used in the diagnosis and management of sepsis. The acute-phase protein C-reactive protein, produced by the liver in response to inflammation, has been the focus of much research due to its potential to identify infection and influence treatment choices [4]. Early detection of sepsis and infectious diseases is frequently aided by the use of PCT, an endogenous peptide released by the C-cells of the thyroid tissue [5,6].

Previous studies have highlighted the individual roles of CRP and PCT in sepsis diagnosis. However, comparative studies, particularly in pediatric populations, are limited [7]. Understanding the relative efficacy of these biomarkers in predicting clinical outcomes is vital for improving sepsis management in children aged 1 month to 15 years [8]. This study aims to evaluate the prognostic utility of CRP and PCT in pediatric sepsis by analyzing key clinical parameters, including the need for ionotropic support, steroid use, the incidence of acute kidney injury (AKI), the requirement for invasive ventilation, and patient outcomes [9]. By comparing these biomarkers, this study seeks to determine their relative effectiveness in predicting severe sepsis complications and guiding clinical interventions in a pediatric intensive care setting. The results will contribute to the expanding body of knowledge on sepsis biomarkers, potentially influencing clinical practice and enhancing outcomes for pediatric sepsis patients.

## Materials and methods

Study design and setting

This prospective study was conducted in the Pediatric Intensive Care Unit (PICU) at Dr. D.Y. Patil Medical College, Hospital, and Research Centre, Pune, spanning from June 2022 to July 2024. Ethical approval for the study was obtained from the institutional review board (I.E.S.C./290/2022) prior to the commencement of the research. A total of 248 children aged one month to fifteen years were enrolled, all diagnosed with sepsis based on the qSOFA score criteria. Exclusion criteria included children with non-infective inflammatory conditions (e.g., rheumatoid arthritis, inflammatory bowel disease), malignancies, or nosocomial infections.

Sample size

A sample size of 248 participants was calculated, with 124 people assigned to each of the two groups (procalcitonin and CRP). Based on a 95% confidence interval, a power of 80%, a significance level of 5%, and a margin of error of ±7.633 standard deviations of the mean, the sample size was determined using the formula

*n* = [2(σ^2^)(*Z*_*α*/2_​+*Z_β​_*)^2​^]/*d*^2^

where n = sample size; σ = standard deviation; *Z*_*α*/2_ = *Z* value for the desired confidence level (in our data, 1.96 for 95% confidence); *Z_β_ *= *Z* value corresponding to the desired power (0.84 for 80% power); *d* = the margin of error.

Participation criteria

Children aged between 1 month and 15 years were included in the study if they were admitted to the PICU and sepsis was diagnosed clinically. Children presenting with non-infective conditions known to influence levels of inflammatory markers were excluded. These conditions included rheumatoid arthritis, inflammatory bowel disease, Wilson’s disease, chronic kidney disease, and multisystem inflammatory syndrome in children. Additionally, children diagnosed with malignancies or nosocomial infections were excluded from the study.

Data collection and biomarker measurement

Upon admission, levels of C-reactive protein and procalcitonin were quantified using the Abbott ARCHITECT system, with follow-up measurements taken at 72 hours post-admission to assess biomarker trends. CRP-positive sepsis was considered with values over 10 mg/dl and procalcitonin-positive with values above 3.0 ng/ml. Comprehensive clinical data were also recorded, encompassing variables such as the need for ionotropic support, steroid use, incidence of AKI, requirement for invasive ventilation, length of PICU stay, and patient outcomes.

Statistical analysis

Data analysis was performed using SPSS statistical software (IBM Corp., Armonk, NY). Categorical data were evaluated using the chi-square test. A significance level of less than 0.05 was employed to determine statistical significance, ensuring robustness in interpreting study findings and their implications for clinical practice.

## Results

In this study, demographic data and initial biomarker measurements were carefully analyzed. The gender distribution was relatively balanced across both groups, with 56 (23%) females and 68 (27%) males in the C-reactive protein group, and 57 (23%) females and 67 (27%) males in the procalcitonin group, showing no significant difference. Mean qSOFA scores, a measure of severity in sepsis, were comparable between the groups (10.64 ± 1.86 in C-reactive protein vs. 10.8 ± 1.97 in procalcitonin, p = 0.54).

However, notable differences emerged in biomarker levels. Patients in the procalcitonin group exhibited significantly lower systolic blood pressure (91.51 ± 12.86 mmHg) compared to those in the C-reactive protein group (95.47 ± 11.59 mmHg, p = 0.012). Furthermore, higher mean levels of serum bilirubin (0.94 ± 0.45 mg/dL vs. 0.54 ± 0.36 mg/dL, p = 0.001) and serum creatinine (1.10 ± 1.02 mg/dL vs. 0.60 ± 1.00 mg/dL, p = 0.0058) were observed in the procalcitonin group compared to the C-reactive protein group. Conversely, there was no significant difference in hemoglobin levels between the groups (10.05 ± 1.87 g/dL vs. 9.98 ± 1.67 g/dL, p = 0.75) (Table 1).

**Table 1:** Demographics and initial biomarker levels

Demographics and biomarker levels	C-reactive protein (n=124)	Procalcitonin (n=124)	p-value
Female	56 (23%)	57 (23%)	-
Male	68 (27%)	67 (27%)	-
qSOFA score (mean ± SD)	10.64 ± 1.86	10.8 ± 1.97	0.54
Systolic BP (mean ± SD) mmHg	95.47 ± 11.59	91.51 ± 12.86	0.012
Serum bilirubin (,mean ± SD) mg/dL	0.54 ± 0.36	0.94 ± 0.45	0.001
Serum creatinine (mean ± SD) mg/dL	0.60 ± 1.00	1.10 ± 1.02	0.0058
Hemoglobin (mean ± SD) g/dL	10.05 ± 1.87	9.98 ± 1.67	0.75

We further did a comparative analysis of interventions and outcomes between two groups of patients, one with elevated CRP levels and the other with elevated procalcitonin levels, each consisting of 124 individuals. In terms of inotrope usage, there was a significant difference between the two groups. In the CRP group, 85 (69%) patients did not receive inotropes, whereas 39 (31%) did. In contrast, in the procalcitonin group, only 47 (38%) patients did not receive inotropes, while 77 (62%) received them, indicating a much higher usage of inotropes among patients with elevated procalcitonin levels. This suggests that patients with higher procalcitonin levels might require more intensive cardiovascular support. Steroid use also varied significantly between the groups. In the CRP group, 114 (92%) patients did not receive and 10 (8%) received the steroids, while in the procalcitonin group, 98 (79%) did not receive steroids and 26 (21%) did. This indicates a higher propensity for steroid administration in the procalcitonin group, possibly reflecting a greater inflammatory response or severity of illness in these patients.

The use of intravenous immunoglobulin (IVIG) did not differ significantly between the two groups, with almost all patients in both groups not receiving IVIG (122, 98% in the CRP group and 121, 98% in the procalcitonin group) and only a small number in each group receiving it (2, 2% in the CRP group and 3, 2% in the procalcitonin group). This suggests that IVIG usage was relatively low and consistent across both groups. Lastly, the occurrence of AKI showed a significant difference. In the CRP group, 113 (91%) patients did not develop AKI, while 11 (9%) did. In the procalcitonin group, 100 (81%) patients did not develop AKI, but 24 (19%) did, indicating a higher incidence of AKI in the procalcitonin group. This could imply a correlation between elevated procalcitonin levels and an increased risk of kidney dysfunction (Table 2).

**Table 2:** Clinical interventions and outcomes

Clinical interventions and outcomes	C-reactive protein (n=124)	Percentage	Procalcitonin (n=124)	Percentage
Inotropes				
No	85	69%	47	38%
Yes	39	31%	77	62%
Steroids				
No	114	92%	98	79%
Yes	10	8%	26	21%
IVIG				
No	122	98%	121	98%
Yes	2	2%	3	2%
AKI				
No	113	91%	100	81%
Yes	11	9%	24	19%

We also compared the type of ventilation methods and the length of the PICU stay between patients with elevated CRP levels and those with elevated procalcitonin levels (Table 3). Regarding ventilation methods, there was a significant difference in the use of invasive versus non-invasive ventilation between the two groups. In the CRP group, 22 patients (18%) required invasive ventilation, while 102 patients (82%) were managed with non-invasive ventilation. In contrast, in the procalcitonin group, 39 patients (31%) needed invasive ventilation, and 85 patients (69%) received non-invasive ventilation. This indicates a higher need for invasive ventilation among patients with elevated procalcitonin levels, suggesting that these patients may have more severe respiratory issues requiring more intensive support.

**Table 3:** Ventilation and length of PICU stay PICU: pediatric intensive care unit.

Ventilation and PICU stay	C-reactive protein (n=124)	Percentage	Procalcitonin (n=124)	Percentage
Ventilation				
Invasive	22	18%	39	31%
Non-invasive	102	82%	85	69%
Length of PICU stay				
1–10 days	103	83%	105	85%
>10 days	21	17%	19	15%

The length of the PICU stay did not show a significant difference between the two groups. In the CRP group, 103 patients (83%) had a PICU stay of 1-10 days, while 21 patients (17%) had a stay of more than 10 days. Similarly, in the procalcitonin group, 105 patients (85%) stayed in the PICU for 1-10 days, and 19 patients (15%) stayed for more than 10 days. This suggests that the duration of PICU stay is comparable between patients with elevated CRP and those with elevated procalcitonin levels, regardless of the differences in the severity of their conditions as indicated by the need for invasive ventilation (Table 3).

We further examined the blood culture results, patient outcomes, and antibiotic management for the two groups of patients. There was no significant difference in blood culture results between the two groups. In the CRP group, 121 (98%) patients showed no bacterial growth in their blood cultures, while 3 (2%) had positive growth. Similarly, in the procalcitonin group, 120 (97%) patients had no bacterial growth, and 4 (3%) had positive growth. This indicates that the incidence of positive blood cultures is low and comparable between the two groups.

The outcomes, in terms of discharge rates and mortality, also do not show a significant difference between the groups. In the CRP group, 115 (93%) patients were discharged, and 9 (7%) patients died. In the procalcitonin group, 111 (90%) patients were discharged, and 13 (10%) patients died. Although the procalcitonin group had a slightly higher mortality rate, the difference is not significant, suggesting that overall outcomes are similar between the two groups.

Regarding antibiotic management, 67 patients (54%) in the prolactin group had their antibiotic treatment downgraded, while 33 patients (46%) had their treatment upgraded. This suggests that a significant proportion of patients with elevated procalcitonin levels had their antibiotic therapy adjusted based on clinical progress or further diagnostic information (Table 4).

**Table 4:** Blood culture, outcomes, and antibiotic management

Blood culture, outcomes, and antibiotic management	C-reactive protein (n=124)	Percentage	Procalcitonin (n=124)	Percentage
Blood culture				
No growth	121	98%	120	97%
Positive growth	3	2%	4	3%
Outcome				
Discharged	115	93%	111	90%
Mortality	9	7%	13	10%
Antibiotic management				
Downgrade	-	-	67	54%
Upgrade	-	-	33	27%

**Figure 1:**
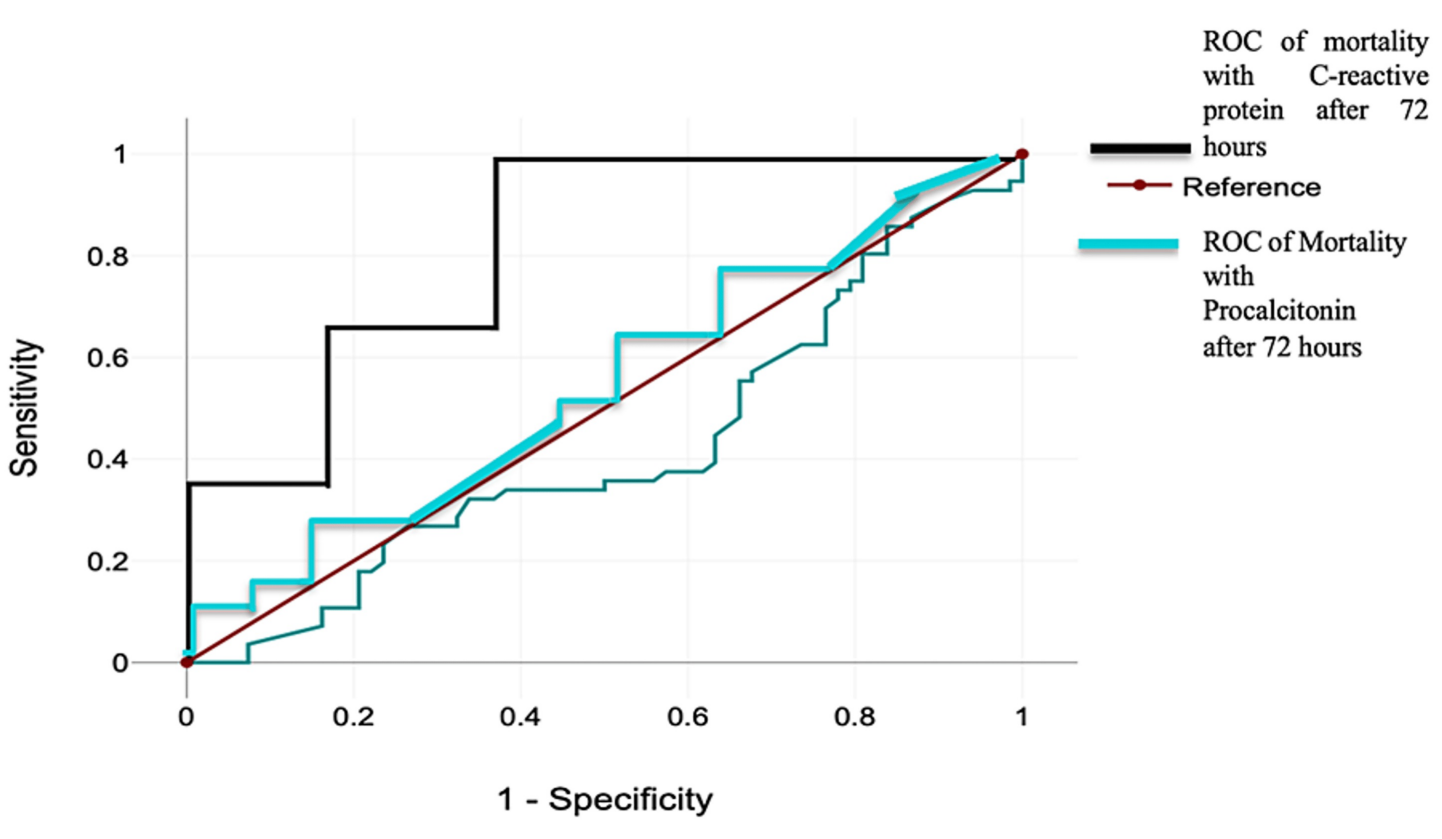
Analysis of ROC curves of correlation ROC: receiver operating characteristic.

Table 5 (Figure 1) provides insights into the diagnostic performance of procalcitonin and CRP measured at day 0 and after 72 hours. On day 0, procalcitonin demonstrates moderate diagnostic ability with an area under the curve (AUC) of 0.604, coupled with high sensitivity (81.8%) and specificity (80.8%) and a significant p-value (≤0.001), indicating its potential as a useful early diagnostic marker. However, CRP on day 0, while showing a strong AUC of 0.848, fails to achieve statistical significance (p=0.144) and has lower sensitivity (63.6%) and specificity (61.0%). This suggests that, at the initial measurement, procalcitonin may be more effective than CRP in identifying true positive and true negative cases.

**Table 5:** ROC curve analysis p-value <0.05 is considered statistically significant. ROC: receiver operating characteristic.

Parameter	Area under curve	S.E.	p-value	95% C.I.		Sensitivity (%)	Specificity
				Lower bound	Upper bound		
Procalcitonin (ng/ml) day 0	0.604	0.065	<0.001	0.711	0.966	81.8	80.8
C-reactive protein (ml/L) day 0	0.848	0.099	0.144	0.435	0.824	63.6	61.0
Procalcitonin (ng/ml) after 72 hours	0.993	0.023	<0.001	0.680	0.998	86.2	89.8
C-reactive protein (ml/L) after 72 hours	0.85	0.087	0.156	0.456	0.875	64.7	62.8

After 72 hours, the diagnostic performance of procalcitonin markedly improves, exhibiting an exceptional AUC of 0.993, high sensitivity (86.2%) and specificity (89.8%), and a significant p-value (≤0.001). This indicates that procalcitonin has become a highly reliable marker for diagnosis and monitoring over time. In contrast, CRP measured after 72 hours maintains a strong AUC of 0.850 but still does not achieve statistical significance (p=0.156) and shows relatively lower sensitivity (64.7%) and specificity (62.8%). These findings highlight that, while both markers are useful, procalcitonin, particularly after 72 hours, is a superior biomarker compared to CRP for prognostic purposes.

## Discussion

This study highlights the greater predictive value of procalcitonin over CRP. PCT, compared to CRP, is significantly associated with more severe clinical parameters, such as lower systolic blood pressure, a greater requirement for ionotropic support, and a higher incidence of acute renal injury. These findings align with previous studies demonstrating the reliability of procalcitonin as a biomarker for severe infections. For instance, a study by Schuetz et al. supports the present observations, showing that procalcitonin levels strongly correlate with the severity of sepsis and are effective in guiding therapeutic interventions [10]. Similarly, Bouadma et al. found that procalcitonin-guided therapy reduced antibiotic duration and improved clinical outcomes in septic patients [11]. The higher serum bilirubin and creatinine levels observed in procalcitonin-positive cases indicate potential liver and renal dysfunction, further emphasizing the marker's utility in predicting organ dysfunction. This is consistent with findings by Zhang and Ni, who reported that elevated procalcitonin levels are predictive of renal and hepatic complications in sepsis [12].

While CRP remains a valuable marker due to its broader availability and cost-effectiveness, it does not exhibit the same level of association with severe clinical parameters as procalcitonin. This study aligns with the results from Jong et al., which concluded that procalcitonin is a more reliable marker for predicting severe complications and guiding intensive care needs [13]. Interestingly, no significant difference was found in the length of PICU stay or patient outcomes between the CRP and procalcitonin groups. This suggests that while procalcitonin may be effective in identifying severe cases and guiding immediate therapeutic interventions, the overall duration of care and outcomes can be affected by various factors beyond just biomarker levels. Studies by Kramer et al. and Schuetz et al. also highlight the multifactorial nature of sepsis management, where comprehensive clinical care is pivotal [14,15].

Furthermore, the present study highlights the role of procalcitonin in antibiotic stewardship. Procalcitonin-guided adjustments led to a significant number of downgrades in antibiotic therapy. In the study, we successfully downgraded antibiotics in 67 patients who exhibited a decrease in PCT levels from day 0 to day 3 (72 hours). This was consistent with the findings of Eichberger et al., who emphasized the importance of procalcitonin in optimizing antibiotic use and reducing unnecessary exposure [16].

## Conclusions

This study underscores the superiority of procalcitonin over CRP as a predictive indicator for pediatric sepsis, particularly in assessing the severity and clinical management needs. Procalcitonin's significant correlation with severe clinical parameters highlights its pivotal role in the early detection and treatment of severe sepsis. It is an effective marker for guiding therapeutic interventions, predicting complications such as organ dysfunction, and determining the necessity for critical care. While CRP remains useful and widely available, procalcitonin's enhanced reliability makes it a better choice for predicting severe sepsis outcomes. These limitations underscore the need for larger, multi-center studies to validate and expand upon these initial findings.
